# Supplementary material for: Quadrupolar magnetic excitations in an isotropic spin-1 antiferromagnet
Source: Nat Commun. 2022 Apr 28;13:2327. doi: 10.1038/s41467-022-30065-5 (PMC9051120; doi:10.1038/s41467-022-30065-5)
Supplement: Supplementary file 1 — Supplementary Information [file 41467_2022_30065_MOESM1_ESM.pdf]

Supplementary Information for "Quadrupolar magnetic excitations in an isotropic spin-1 antiferromagnet"

Supplementary Note 1: Experimental details

The single crystal of Y_2BaNiO_5 was pre-aligned using Laue diffraction so that its chain direction lies in the RIXS scattering plane (Fig. 1a). The momentum transfer resolution is less than 0.011 $(2\pi/c)$ (see Fig. 1b). Negative and positive values of $q_{||}$ represent x-ray grazing-incident and grazing-exit geometries, respectively. The strongest specular elastic reflection was seen close to $q_{||} = -0.03$, as shown in Fig. 2(a) of main text. This position originates from a combination of the high quality mirror-like cleaved surface of the crystal and the arrangement of optical elements used for collecting the scattered x-rays (see Fig. 1c, d).

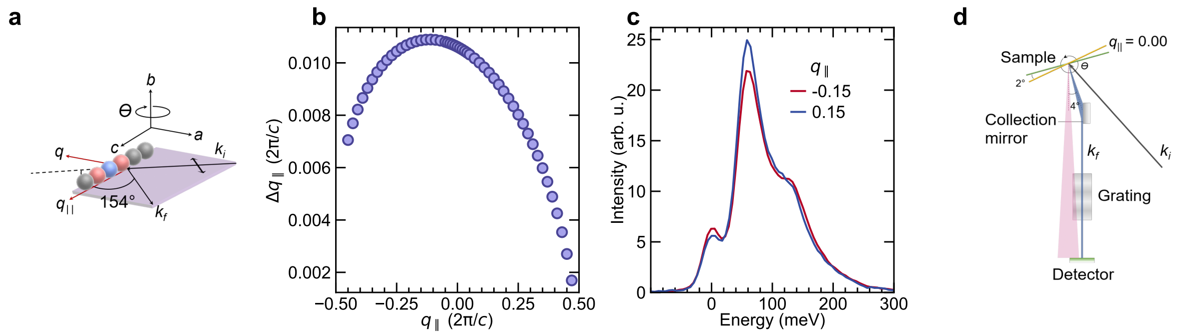

Supplementary Figure 1. **a** RIXS geometry for probing spin excitations in Y_2BaNiO_5. Throughout the paper, projection of momentum transfer ($q_{||}$) in units of $2\pi/c$ along the chain direction is presented. **b** The momentum transfer resolution for our experimental setup as a function of $q_{||}$. **c** A comparison of the RIXS spectra recorded at $\pm q_{||}$. The agreement between the two RIXS spectra confirm that our assigned $q_{||} = 0.00$ corresponds to the true specular reflection condition. **d** A schematic representation of the optical arrangement for scattered X-rays from sample at I21 RIXS beamline, Diamond Light Source. The k_f and true momentum transfer values are determined by the scattered beam collected by a plane parabolic mirror $\sim 4°$ higher than the direct scattered beam direction. The incidence angle on the sample for specular reflection condition for the direct beam is, therefore, $\sim 2°$ lower than that for the true specular reflection condition at $q_{||} = 0.00$. Usually this direct beam is weak and does not reach the detector. However, due to the high quality mirror like cleaved surface of the crystal, a strong specular elastic reflection corresponding to this direct beam is seen close to $q_{||} = -0.03$ in Fig. 2(a) of the main text.

Supplementary Note 2: RIXS data fitting

Fig. 2 shows the extracted values of coefficients C_0, C_1 and C_2 of the three different types of excitations considered for fitting the RIXS data as described in Methods of main text. Fig. 3

shows the fit profiles of the different components. Fig. 4 shows the fitting of the $\Delta S_{\rm tot} = 1$ triplet excitations at $q_{\|} = 0.47$.

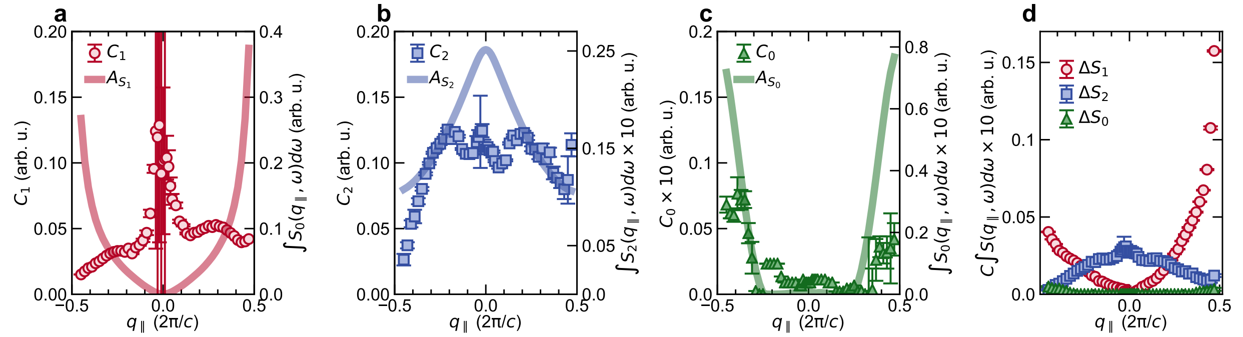

Supplementary Figure 2. **a, b, c** Coefficients C_1, C_2, and C_0 accounting for changes in the RIXS scattering cross section with varying x-ray incidence for ΔS_1, ΔS_2, and ΔS_0 excitations, respectively. The coefficient values are determined by fitting RIXS spectra with dynamical spin susceptibilites obtained from DMRG using Eq. (5) given in Methods. Error bars are least square fit errors. Also shown by the continuous lines are the integrated dynamical spin structure factors $A_S(q_{\|}) = \int S(q_{\|}, \omega) d\omega$ from DMRG for the ΔS_1, ΔS_2, and ΔS_0 excitations. **d** Total RIXS intensities for ΔS_1, ΔS_2, and ΔS_0 excitations.

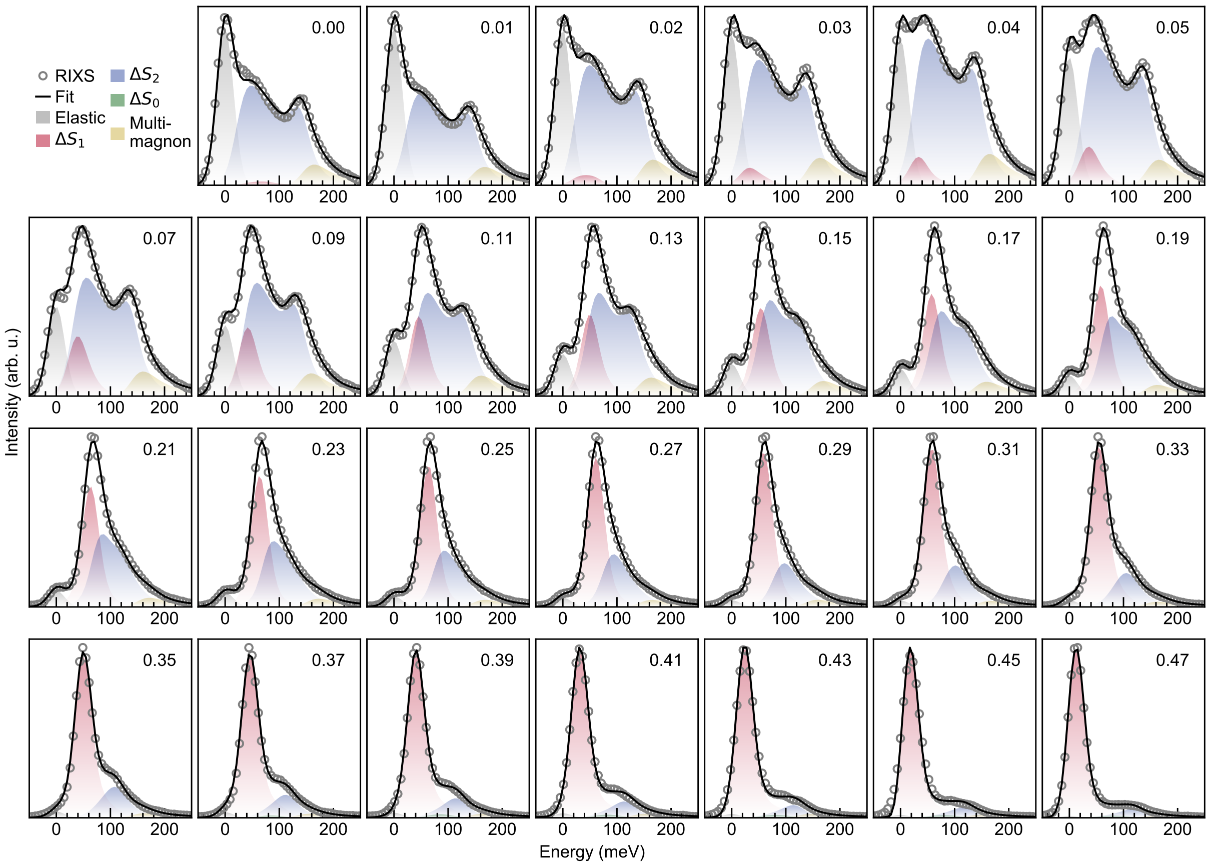

Supplementary Figure 3. Ni L-edge RIXS line spectra from Y_2BaNiO_5 at 11 K, with fits derived from the spin susceptibilities obtained from DMRG using Eq. (5) given in Methods. Corresponding $q_{\|}$ value is written in each panel.

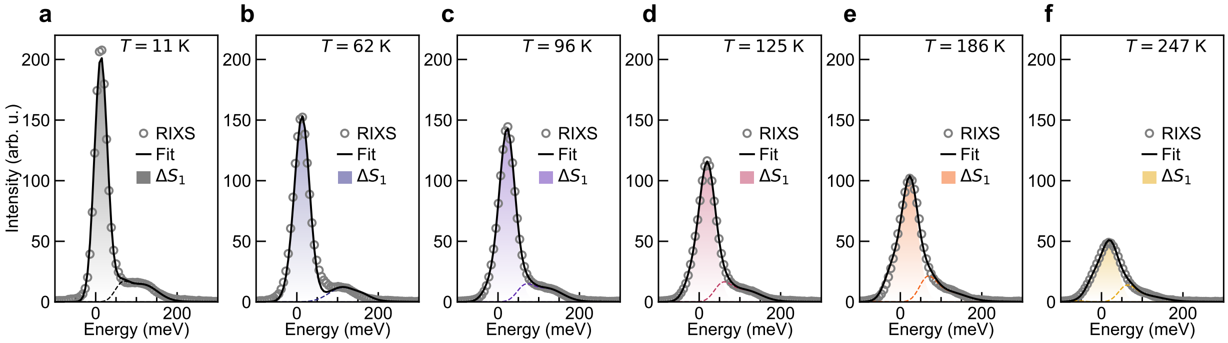

Supplementary Figure 4. **a-f** Temperature dependent RIXS spectra at $q_{\|} = 0.47$. Shaded regions are fits to $\Delta S_{\mathrm{tot}} = 1$ triplet excitations using resolution-convoluted damped harmonic oscillator functions weighted by the Bose factor. The extracted amplitudes have been used in Fig. 4d of main text. The dashed lines indicate contributions from the $\Delta S_{\mathrm{tot}} = 2$ and multi-magnon excitations.

Supplementary Note 3: Influence of temperature on the boundaries of two-ΔS_1 excitations.

In a simple picture, if one considers a continuum from pairs of non-interacting ΔS_1 triplets due to single spin-flips at multiple sites, then bandwidth reduction of each would manifest as the overall raising and lowering of the lower and upper boundaries of the continuum, respectively. In Fig. 5a and b, we show the change of such continuum boundaries upon changing the Haldane gap energy Δ_H using the semi-quantitative Haldane dispersion relation given in the caption of Fig. 2 of main text for two ΔS_1 triplets. In Fig. 5c and d, we show the change of the continuum boundaries upon changing the velocity v of the ΔS_1 triplets and in Fig. 5e and f, the combined effect of Δ_H and v. As temperature is raised, due to the increase (decrease) of the Δ_H (v), the energy of the lower (upper) boundary of the continuum at $q_{\|} = 0$ increases (decreases). Similar effect is seen on the two components of the ΔS_2 excitations in Fig. 4 of main text.

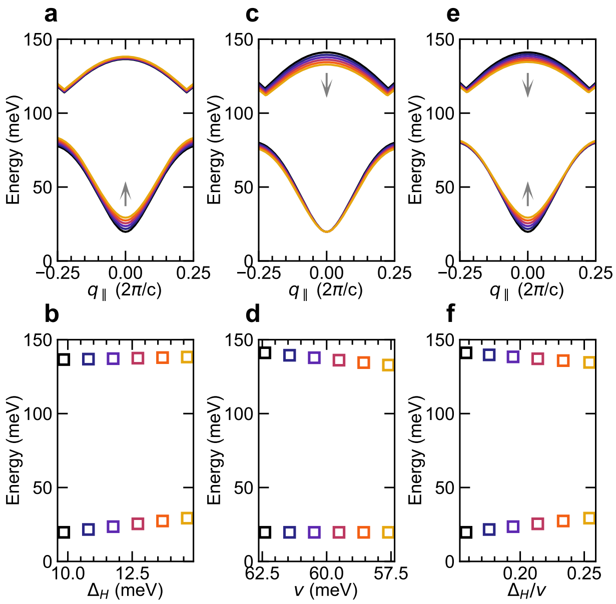

Supplementary Figure 5. **a**, The influence of Haldane gap Δ_H on the boundaries of two-ΔS_1 excitations. The arrow indicates the hardening of the lower boundary with increasing Δ_H. **b,** The variation in the energies of the upper and lower boundaries at $q_{\|} = 0$ with increasing Δ_H extracted from the curves in panel **a**. **c** The influence of the velocity v of the ΔS_1 excitations on the boundaries of two-ΔS_1 excitations. The arrow indicates the softening of the upper boundary with decreasing v. **d,** The variation in the energies of the upper and lower boundaries at $q_{\|} = 0$ with decreasing v extracted from the curves in panel **c**. **e** The influence of Δ_H and v on the boundaries of the two ΔS_1 excitations. The arrow indicates the hardening (softening) of the lower (upper) boundary with increasing (decreasing) Δ_H (v). **f,** The variation in the energies of the upper and lower boundaries at $q_{\|} = 0$ with increasing (decreasing) Δ_H (v), extracted from the corresponding curves in panel **e**.

Supplementary Note 4: Real-space representations for the dynamical structure factors

Here, we provide the real-space representation of the dynamical structure factors studied in the main text. For a finite system, the dynamical structure factor is given by

$$S_\alpha(q_\parallel,\omega) = \frac{1}{L}\sum_{j=1}^{L} e^{\mathrm{i}q_\parallel(j-c)}\Big\{-\frac{1}{\pi}\mathrm{Im}\Big[\langle\psi|S_j^\alpha \frac{1}{\omega - H + \mathrm{i}\eta} S_c^\alpha|\psi\rangle\Big]\Big\}, \tag{1}$$

where the operator S_j^α is applied at the center $j = c$ of the long chains with open boundaries[1–3], and $|\psi\rangle$ represents the ground state of the system.

Using the Liouville[4] and the purification approaches[5–7], the dynamical structure factor in real space and a finite temperature $T = 1/\beta$ is

$$S_\alpha(q_\parallel,\omega) = \frac{1}{L}\sum_{j=1}^{L} e^{\mathrm{i}q_\parallel(j-c)}\Big\{-\frac{1}{\pi}\mathrm{Im}\Big[\langle\psi(\beta)|S_j^\alpha \frac{1}{\omega - \mathcal{L} + \mathrm{i}\eta} S_c^\alpha|\psi(\beta)\rangle\Big]\Big\}, \tag{2}$$

where $\mathcal{L} = H \otimes I + I \otimes (-H)$ is the Liouville operator and $|\psi(\beta)\rangle$ is the purified thermal state given by $|\psi(\beta)\rangle = e^{-\beta(H\otimes I)/2}|\psi_\infty\rangle$. Here, the Hamiltonian acts only on the physical sites of the system and $|\psi_\infty\rangle$ is the maximally entangled infinite temperature state (more details are provided in the Supplementary Note 4).

Supplementary Note 5: Magnon-like states unveiled by the dynamical spin correlations in Haldane spin chains

In this section we analyze the excitations encoded in the three dynamical correlation functions introduced in the main text in Eq. (1) in terms of single triplet excitations or *magnon* states in the Haldane chain. Following Eq. (5) in Ref. [8], a single magnon state in an infinite chain with momentum $q_\parallel$ and spin quantum number $S^z = \alpha$ has the form

$$M_{q_\parallel,\alpha}|\psi\rangle = |q_\parallel,\alpha\rangle = \sum_j e^{\mathrm{i}q_\parallel j} c_j^\dagger(q_\parallel,\alpha)|\psi\rangle, \tag{3}$$

where the operator $c_j^\dagger(q_\parallel,\alpha)$ includes the S_j^+ operator and products of multiple spin operators in the vicinity of site j. In other words, single magnons are not constructed by applying only local spin operators $S_{q_\parallel}^1 = \frac{1}{\sqrt{L}}\sum_j e^{\mathrm{i}q_\parallel j} S_j^+$. When $S_{q_\parallel}^1$ is applied to the ground state, the resulting state

can be expressed at low energy by a multi-magnon expansion

$$S^1_{q_\parallel}|\psi\rangle = \Big(\sum_{q_\parallel,\alpha} t^1(q_\parallel,\alpha)M_{q_\parallel,\alpha} + \sum_{q^1_\parallel,\beta,q^{(2)}_\parallel,\gamma} s^1(q^{(1)}_\parallel,\beta,q^{(2)}_\parallel,\gamma)M_{q^{(1)}_\parallel,\beta}M_{q^{(2)}_\parallel,\gamma} + ...\Big)|\psi\rangle, \tag{4}$$

where two-magnon and higher-order multi-magnon states naturally appear. Note that $t^1(q_\parallel,\alpha)$ and $s^1(q^{(1)}_\parallel,\beta,q^{(2)}_\parallel,\gamma)$ encapsulate the projection of the $S^1_{q_\parallel}|\psi\rangle$ state on the single- and two-magnon wave functions, respectively. The expression above tells us that the dynamical correlation function $S_1(q_\parallel,\omega)$ encodes information about single magnon excitations, but also two-magnon and three-magnon excitations as well [9, 10] (with a reduced spectral weight). By symmetry arguments, it is easy to prove that $S^0_{q_\parallel}$ and $S^2_{q_\parallel}$ operators can only induce states with single or multiple pairs of magnon excitations

$$S^0_{q_\parallel}|\psi\rangle = \Big(t^0(q_\parallel) + \sum_{q^1_\parallel,\beta,q^{(2)}_\parallel,\gamma} s^0(q^{(1)}_\parallel,\beta,q^{(2)}_\parallel,\gamma)M_{q^{(1)}_\parallel,\beta}M_{q^{(2)}_\parallel,\gamma} + ...\Big)|\psi\rangle, \tag{5}$$

$$S^2_{q_\parallel}|\psi\rangle = \Big(\sum_{q^1_\parallel,\beta,q^{(2)}_\parallel,\gamma} s^2(q^{(1)}_\parallel,\beta,q^{(2)}_\parallel,\gamma)M_{q^{(1)}_\parallel,\beta}M_{q^{(2)}_\parallel,\gamma} + ...\Big)|\psi\rangle, \tag{6}$$

Here, $s^{0(2)}(q^{(1)}_\parallel,\beta,q^{(2)}_\parallel,\gamma)$ describes the projection of the $S^{0(2)}_{q_\parallel}|\psi\rangle$ states on the two-magnon wave function.

Note that the dynamical correlator $S_0(q_\parallel,\omega)$ defined in the main text naturally contains an elastic peak since the $S^0_{q_\parallel}$ operator is equal to the Hamiltonian operator at $q_\parallel = 0$ and $t^0(q_\parallel = 0) = E_{\rm gs}$.

Supplementary Note 6: Bound state of ΔS_2 excitations

In this section, we discuss the physical origin of the sharp peak observed in the ΔS_2 spectrum close to $q_{\|} = 0$ with an energy of $\simeq 136$ meV (see Figs. 2e and 2f of the main text).

In low-dimensional systems, a van Hove singularity in magnetic density of states can give rise to sharp peaks at momentum values where the magnetic dispersion has stationary points. The sharp peaks observed in our study could therefore originate either from a van Hove singularity in the triplet density of states, or from the formation of a bound state of the triplet pairs *above* the upper boundary of the continuum formed (equivalent to two-magnons).

Adopting the dispersion of ΔS_1 triplet excitations [11–14] obtained from our DMRG calculations $\omega^2_{S_1}(q_{\|}) = \Delta^2_{\mathrm{H}} + v^2\sin^2 q_{\|} + \alpha^2\cos^2\frac{q_{\|}}{2}$, stationary points are located at $q_{\|} = 0$ and $\simeq 0.25$ $(2\pi/c)$ (see Fig. 6d). At $q_{\|} \simeq 0.25$, where the ΔS_1 excitation has the highest energy and thus can contribute to the highest triplet pair energy, the ΔS_1 density of states has a square-root singularity $\sim (|\omega - \omega_{S_1}(q_{\|} \simeq 0.25)|)^{-1/2}$. Ignoring matrix elements effects, the triplet pair density of states can be assumed to be proportional to the *joint* ΔS_1 density of states $\rho_2(q_{\|}, \omega) = \sum_{k_{\|}} \delta(\omega - \omega_{S_1}(k_{\|}) - \omega_{S_1}(q_{\|} - k_{\|}))$, which is shown in Fig. 6b. Fig. 6c shows the DMRG calculated $S_2(q_{\|} = 0, \omega)$ with the sharp peak at an energy of ~ 136 meV. Compared to it, $\rho_2(q_{\|} = 0, \omega)$ has an asymmetric peak corresponding to the van Hove singularity at ~ 125 eV (lower by ~ 11 meV). Evidently, the sharp component of the ΔS_2 excitation does not arise from the singularities in the magnetic density of states if the triplets are non-interacting and therefore have negligible bandwidth renormalisation.

In Fig. 6c, $S_1(q_{\|} = 0.25, 2\omega)$ is plotted by doubling the energy scale, to show that the maximum energy of a pair of non-interacting triplets can be 129 meV ($\sim 64.5 \times 2$, the true energy scale for $S_1(q_{\|} = 0.25, \omega)$ is given on top of the panel). The sharp peak obtained in the ΔS_2 excitations is therefore ~ 7 meV higher than the non-interacting scenario at $q_{\|} = 0$. Also, shown in Fig. 6a are the lower and upper boundaries of the continuum from pairs of non-interacting triplets (equivalent to the two-magnon continuum). There, the sharp peaks for the ΔS_2 excitation appear above the upper boundary of the continuum throughout the probed region of reciprocal space. The small positive value therefore suggests a weak *repulsive* interaction between the triplets formed after a quadrupolar ΔS_2 excitation [8], and the sharpness of the peaks suggests that a bound state of the triplet pairs forms.

We here provide a semi-quantitative analysis to show that at sufficiently high energies, a pair of triplet excitations form a bound state above the two-triplet continuum in presence of weak repulsion between them. The ΔS_1 dispersion can be expanded around $q_{\|} \simeq \pm 0.25$, which are the

likely momentum states contributing to the formation of the highest energy triplet pair state at $q_\parallel \simeq 0$. From Fig. 6d, we obtain

$$E(q_\parallel \simeq \pm 0.25 + \delta q_\parallel) \simeq \omega_{S_1}(q_\parallel = \pm 0.25) + \frac{\hbar^2 (\delta q_\parallel)^2}{2m^*}, \tag{7}$$

A Schrödinger equation can thus be set up for these two quasiparticles resulting from independent ΔS_1 excitations:

$$\Big[-\frac{\hbar^2}{2m^*}\frac{\partial^2}{\partial r_1^2} - \frac{\hbar^2}{2m^*}\frac{\partial^2}{\partial r_2^2} + V(r_1, r_2)\Big]\Psi(r_1, r_2) = (E - 2\omega_{S_1}(q_\parallel \simeq 0.25))\Psi(r_1, r_2), \tag{8}$$

Since we are interested in the triplet pair state at $q_\parallel \simeq 0$, we can neglect the center of mass motion ($\delta q_\parallel^{\rm cm} = \delta q_\parallel = 0$ and $E = \tilde{E} + \frac{\hbar^2 (\delta q_\parallel)^2}{2m^*_{cm}} = \tilde{E}$) and consider just the dynamics in the relative coordinate $r = r_1 - r_2$:

$$\Big[-\frac{\hbar^2}{2m^*}\frac{\partial^2}{\partial r^2} + V(r)\Big]\Psi(r) = (\tilde{E} - 2\omega_{S_1}(q_\parallel \simeq 0.25))\Psi(r), \tag{9}$$

Assuming a repulsive δ-like potential among the $\Delta S_{tot} = 2$ excitations [8], $V(r) = \lambda\delta(r)$, with $\lambda > 0$ and noting that the effective mass to be negative $m^* = -|m^*|$ we get

$$\Big[-\frac{\hbar^2}{2|m^*|}\frac{\partial^2}{\partial r^2} - \lambda\delta(r)\Big]\Psi(r) = E'\Psi(r), \tag{10}$$

where we have multiplied the equation by -1 and then redefined $E' = -\tilde{E} + 2\omega_{S_1}(q_\parallel \simeq 0.25)$. Elementary quantum mechanics then predicts the existence of a bound state with energy

$$E'_B = -\frac{|m^*|\lambda^2}{2\hbar^2}, \tag{11}$$

Eq. (11) implies the existence of a bound state above the continuum, since for $E' < 0$ we have $\tilde{E} > 2\omega_{S_1}(q_\parallel \simeq 0.25)$, and $\tilde{E} = 2\omega_{S_1}(q_\parallel \simeq 0.25) + |E'_B|$. From this expression, we can *a posteriori* estimate the strength of the quasiparticle interaction λ (which has units of energy times length, so we divide by the lattice unit c) assuming $|E'_B| \simeq 7$ meV:

$$\lambda/c = \sqrt{4\frac{\hbar^2}{2|m^*|}|E'_B|} \simeq 39 \text{ meV} \simeq 1.6J, \tag{12}$$

This is a rather weak interaction if we compare it with the ΔS_1 bandwidth $W \simeq 2.3J$ and therefore validates our previously estimated energy scales of the triplet pair density of states and continuum

boundaries.

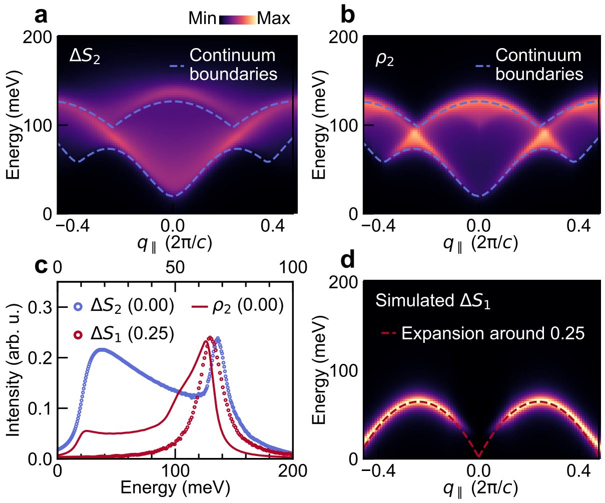

Supplementary Figure 6. **a,** $S_2(q_{||}, \omega)$ from DMRG. **b,** $\rho_2(q_{||}, \omega)$ obtained from joint ΔS_1 density of states. Both panels are over plotted with the lower and upper boundaries of the continuum from pairs of non-interacting triplets (equivalent to the two-magnon continuum). **c,** $S_2(q_{||} = 0, \omega)$, $\rho_2(q_{||} = 0, \omega)$ and $S_1(q_{||} = 0.25, 2\omega)$. The top x-axis is the energy scale for $S_1(q_{||} = 0.25, \omega)$. **d,** One magnon dispersion relationship fitted from DMRG and experimental RIXS data. We expand it around $q_{||} \simeq \pm 0.25$ obtaining free quasiparticles with *negative* mass. Here negative mass just means that the triplet energy cannot be bigger than $\omega_{S_1}(q_{||} \simeq 0.25) = 64.5$ meV.

To further confirm this estimate, we simulated a Bose Hubbard model on a 1D lattice of a finite length with open boundary conditions with an on-site attractive interaction $U_b = -|U_b|$ with $N_b = 2$ bosons

$$H = -t_b \sum_i (b_i^\dagger b_{i+1} + \text{h.c.}) + \frac{U_b}{2} \sum_i n_i^b (n_i^b - 1), \tag{13}$$

We have assumed $t_b = 0.575J$ such that the bandwidth is $W_b = 4t_b = 2.3J$. We find that on a long chain of $L = 64$ sites, the system shows a bound state with energy $E_B = E(U_b = -1.675J) - E(U_b = 0) \simeq -0.28J \sim -6.7$ meV, consistent with our estimation above using an attractive δ-like potential between the quasiparticles.

Supplementary Note 7: Computational details to reproduce the DMRG results

Here we provide instructions on how to reproduce the DMRG results used in the main text. The results reported in this work were obtained with DMRG++ versions 6.01 and PsimagLite versions 3.01. The DMRG++ computer program [15] can be obtained with:

```
git clone https://github.com/g1257/dmrgpp.git
git clone https://github.com/g1257/PsimagLite.git
```

The main dependencies of the code are BOOST and HDF5 libraries. To compile the program:

```
cd PsimagLite/lib; perl configure.pl; make
cd ../../dmrgpp/src; perl configure.pl; make
```

The DMRG++ documentation can be found at `https://g1257.github.io/dmrgPlusPlus/manual.html` or can be obtained by doing `cd dmrgpp/doc; make manual.pdf`. In the description of the DMRG++ inputs below, we follow very closely the description in the supplemental material of Ref. [16], where similar calculations were performed. Other inputs and scripts can be made available upon request.

a) Obtaining zero-temperature spectra

The $T = 0$ results can be reproduced as follows. We first run `./dmrg -f inputGS.ain -p 12` to obtain the ground state wave-function and ground state energy with 12 digit precision using the `-p 12` option. The `inputGS.ain` has the form

```
##Ainur1.0
TotalNumberOfSites=100;
NumberOfTerms=2;

### 1/2(S^+S^- + S^-S^+)
gt0:DegreesOfFreedom=1;
gt0:GeometryKind="chain";
gt0:GeometryOptions="ConstantValues";
gt0:dir0:Connectors=[1.0];

### S^zS^z part
```

```
gt1:DegreesOfFreedom=1;
gt1:GeometryKind="chain";
gt1:GeometryOptions="ConstantValues";
gt1:dir0:Connectors=[1.0];

Model="Heisenberg";
HeisenbergTwiceS=2;
SolverOptions="twositedmrg";
InfiniteLoopKeptStates=100;
FiniteLoops=[[49, 1000, 0],
[-98, 1000, 0],
[98, 1000, 0],
[-98, 1000, 0],
[98, 1000, 0]];

# Keep a maximum of 1000 states, but allow SVD truncation with
# tolerance 1e-10 and minimum states equal to 100
TruncationTolerance="1e-10,100";
# Symmetry sector for ground state S^z_tot=0
TargetSzPlusConst=100
```

Here we showed the input for S=1 (note `HeisenbergTwiceS=2`). The parameter TargetSzPlusConst should be equal $Sz + L$, where Sz is the targeted Sz sector and L is the system size. The next step is to calculate dynamics for the $S^0(q_{\|}, \omega)$, $S^1(q_{\|}, \omega)$ and $S^2(q_{\|}, \omega)$ spectral functions using the saved ground state as an input. For simplicity, we discuss the $S^1(q_{\|}, \omega)$ first. It is convenient to do the dynamics run in a subdirectory `S1`, so `cp inputGS.ain S1/inputS1.ado` and add/modify the following lines in `inputS1.ado`

```
SolverOptions="twositedmrg,restart,minimizeDisk,CorrectionVectorTargeting";
# The finite loops now start from the final loop of the gs calculation
FiniteLoops=[[-98, 2000, 2],
[98, 2000, 2],
[-98, 2000, 2],
[98, 2000, 2]];
```

```
TruncationTolerance="1e-7,100";

# RestartFilename is the name of the GS .hd5 file (extension is not needed)
RestartFilename="../inputGS";

# The weight of the g.s. in the density matrix
GsWeight=0.1;
# Legacy, set to 0
CorrectionA=0;
# Fermion spectra has sign changes in denominator.
# For boson operators (as in here) set it to 0
DynamicDmrgType=0;
# The site(s) where to apply the operator below. Here it is the center site.
TSPSites=[49];
# The delay in loop units before applying the operator. Set to 1
TSPLoops=[1];
# If more than one operator is to be applied, how they should be combined.
# Irrelevant if only one operator is applied, as is the case here.
TSPProductOrSum="sum";
# How the operator to be applied will be specified
string TSPOp0:TSPOperator=expression;
# The operator expression
string TSPOp0:OperatorExpression="splus";
# How is the freq. given in the denominator (Matsubara is the other option)
CorrectionVectorFreqType="Real";
# This is a dollarized input, so the
# omega will change from input to input.
CorrectionVectorOmega=$omega;
# The broadening for the spectrum in omega + i*eta
CorrectionVectorEta=0.25;
# The algorithm
CorrectionVectorAlgorithm="Krylov";
#The labels below are ONLY read by manyOmegas.pl script
```

```
# How many inputs files to create
#OmegaTotal=600
# Which one is the first omega value
#OmegaBegin=-2.0
# Which is the "step" in omega
#OmegaStep=0.025
# Because the script will also be creating the batches,
# indicate what to measure in the batches
#Observable=sminus
```

Then all individual inputs (one per ω in the correction vector approach) can be generated and submitted using the `manyOmegas.pl` script which can be found in the `dmrgpp/src/script` folder:

```
perl manyOmegas.pl inputS1.ado BatchTemplate.pbs <test/submit>.
```

It is recommended to run with `test` first to verify correctness, before running with `submit`. Depending on the machine and scheduler, the `BatchTemplate` can be e.g. a PBS or SLURM script. The key is that it contains a line `./dmrg -f $$input "<X0|$$obs|P2>" -p 12` which allows `manyOmegas.pl` to fill in the appropriate input for each generated job batch. After all outputs have been generated,

```
perl procOmegas.pl -f inputS1.ado -p
perl pgfplot.pl
```

can be used to process and plot the results (these scripts are also given in `dmrgpp/src/script` folder).

For the calculation of the quadrupolar spectral function $S^2(q_\parallel, \omega)$ we would have to substitute in the file above the lines

```
OperatorExpression="splus*splus";
#Observable=sminus*sminus
```

These lines will allow to apply the operator $(S_c^+)^2$ at the center of the chain and to measure its hermitian conjugate $(S_j^-)^2$ on all others sites when we run with `./dmrg -f $$input "<X0|$$obs|P2>"`.

For the calculations of the $S^0(q_\parallel, \omega)$ we would need to perform four separate calculations:

$$S^0_{\text{part}-1}(q_\parallel, \omega) = \frac{1}{L} \sum_{j=1}^{L} e^{iq_\parallel (j-c)} \Big\{ -\frac{1}{\pi} \text{Im} \Big[\langle \psi | S_j^z S_{j+1}^z \frac{1}{\omega - H + i\eta} S_c^z S_{c+1}^z | \psi \rangle \Big] \Big\} \tag{14}$$

$$S^0_{\text{part}-2}(q_\parallel,\omega)=\frac{1}{L}\sum_{j=1}^{L}e^{iq_\parallel(j-c)}\Big\{-\frac{1}{\pi}\text{Im}\Big[\langle\psi|(S^+_jS^-_{j+1}+S^-_jS^+_{j+1})\frac{1}{\omega-H+i\eta}S^z_cS^z_{c+1}|\psi\rangle\Big]\Big\} \tag{15}$$

$$S^0_{\text{part}-3}(q_\parallel,\omega)=\frac{1}{L}\sum_{j=1}^{L}e^{iq_\parallel(j-c)}\Big\{-\frac{1}{2\pi}\text{Im}\Big[\langle\psi|(S^+_jS^-_{j+1}+S^-_jS^+_{j+1})\frac{1}{\omega-H+i\eta}S^+_cS^-_{c+1}|\psi\rangle\Big]\Big\} \tag{16}$$

$$S^0_{\text{part}-4}(q_\parallel,\omega)=\frac{1}{L}\sum_{j=1}^{L}e^{iq_\parallel(j-c)}\Big\{-\frac{1}{2\pi}\text{Im}\Big[\langle\psi|(S^+_jS^-_{j+1}+S^-_jS^+_{j+1})\frac{1}{\omega-H+i\eta}S^-_cS^+_{c+1}|\psi\rangle\Big]\Big\} \tag{17}$$

We should then combine the results as $S^0(q_\parallel,\omega)=S^0_{\text{part}-1}(q_\parallel,\omega)+S^0_{\text{part}-2}(q_\parallel,\omega)+\frac{1}{2}[S^0_{\text{part}-3}(q_\parallel,\omega)+S^0_{\text{part}-4}(q_\parallel,\omega)]$. For simplicity, we here only provide instructions for the calculation of the spectral function $S^0_{\text{part}-1}(q_\parallel,\omega)$. This needs the following modifications in the `inputS0part1.ado`

```
# minimizeDisk option should be removed
SolverOptions="twositedmrg,restart,CorrectionVectorTargeting";
# The finite loops need to have save option 3 at the last loop.
# This is to prepare observation of two-point correlations
FiniteLoops=[[-98, 2000, 2],
[98, 2000, 2],
[-98, 2000, 2],
[98, 2000, 3]];
string TruncationTolerance="1e-7,100";

# The sites where to apply the operator S^{z}_{c}S^{z}_{c+1}.
# Here center and center+1 sites
TSPSites=[49,50];
# The delay in loop units before applying the operator. Set to 0
TSPLoops=[0,0];
TSPProductOrSum="product";
# How the operator to be applied will be specified
string TSPOp0:TSPOperator=expression;
string TSPOp0:OperatorExpression="sz";
string TSPOp1:TSPOperator=expression;
string TSPOp1:OperatorExpression="sz";
```

In this case, the `BatchTemplate` needs to contain the lines running `dmrg` and the `observe` executables

```
./dmrg -f $$input  -p 12
./observe -f $$input -p 12 "<gs|sz;sz|P2>"
```

Straightforward modifications of the script `manyOmegas.pl` need to performed to gather the resulting real space correlations and their Fourier Transform. Input files for the calculation of $S^0_{\mathrm{part}-2}(q_\parallel,\omega)$, $S^0_{\mathrm{part}-3}(q_\parallel,\omega)$ and $S^0_{\mathrm{part}-4}(q_\parallel,\omega)$ and associated scripts for processing the numerical results can be provided upon request.

b) Obtaining finite-temperature spectra

As noted in the Methods section in the main text, finite temperature $T > 0$ calculations proceed in three steps (see also Refs. [4, 5]): We first prepare the system in a $T \to \infty$ (inverse temperature $\beta = 1/T = 0$) state using a fictitious "entangler" Hamiltonian H_E acting in an enlarged Hilbert space. In the purification approach, the geometry of the system can be described as a spin ladder, with physical sites on one leg (with even sites 0,2,4,...) and ancilla sites on the other (with odd sites 1,3,5,...).

The entangler Hamiltonian is chosen such that its ground state $|\psi_\infty\rangle$ corresponds to the $T \to \infty$ state of the physical system when the ancilla degrees of freedom are traced out, $\rho = \mathrm{Tr}\,[|\psi_\infty\rangle\langle\psi_\infty|]_A$. We use a conventional entangler Hamiltonian H_E conserving only the total magnetization of the enlarged physical+ancilla system.

We find the ground state of H_E by running `./dmrg -f Entangler.ain -p 12`, where `Entangler.ain` for L=32 sites (32 physical and 32 ancillas) is given by

```
##Ainur1.0
TotalNumberOfSites=64;
NumberOfTerms=2;
gt0:DegreesOfFreedom=1;
gt0:GeometryKind="ladder";
gt0:LadderLeg=2;
gt0:GeometryOptions="ConstantValues";
gt0:dir0:Connectors=[0.0];
gt0:dir1:Connectors=[-1.0];

gt1:DegreesOfFreedom=1;
gt1:GeometryKind="chain";
```

```
gt1:GeometryOptions="ConstantValues";
gt1:dir0:Connectors=[0];

Model="Heisenberg";
integer HeisenbergTwiceS=2;
SolverOptions="twositedmrg,MatrixVectorOnTheFly";
InfiniteLoopKeptStates=100;
FiniteLoops=[[ 31, 1000, 0],
[-62, 1000, 0],
[ 62, 1000, 0],
[-62, 1000, 0]];
# Keep a maximum of 1000 states, but allow
# density matrix truncation with tolerance and minimum states as below
TruncationTolerance="1e-8,100";
TargetSzPlusConst=64
```

Second, the system is cooled through evolving in imaginary time with the physical Hamiltonian H acting only on physical sites $e^{-\beta(H\otimes I)/2}$, where I is the identity operator in the ancilla space. To compute the thermal state of the system $|\psi(\beta)\rangle = e^{-\beta(H\otimes I)/2}|\psi_\infty\rangle$, we used the Krylov algorithm for time evolution with an imaginary time step $\Delta\beta = 0.025/J$. The time evolution is done with an evolution operator $exp[-\beta' H/2]$ where we define $\beta' = \beta/2$ in units of $1/J$.

Technically, we compute the imaginary time evolution with `./dmrg -f EvolutionT.ain -p 12`, where `EvolutionT.ain` reads (we report only the main differences with respect to the previous input)

```
gt0:GeometryKind="ladder";
gt0:LadderLeg=2;
gt0:GeometryOptions="none";
gt0:dir0:Connectors=[1.0, 0.0, 1.0, 0.0, 1.0, 0.0, 1.0, 0.0, ...];
gt0:dir1:Connectors=[0.0, 0.0, 0.0, 0.0, ...];

gt1:GeometryKind="ladder";
gt1:LadderLeg=2;
gt1:GeometryOptions="none";
```

```
gt1:dir0:Connectors=[1.0, 0.0, 1.0, 0.0, 1.0, 0.0, 1.0, 0.0, ...];
gt1:dir1:Connectors=[0.0, 0.0, 0.0, 0.0, ...];

string PrintHamiltonianAverage="s==c";
string RecoverySave="@M=100,@keep,1==1";
SolverOptions="twositedmrg,restart,TargetingAncilla";

# Notice the save option 3 at the last loop corresponding to the
# physical temperature of interest (in this specific case 250K)
# This allows to measure static correlation functions
FiniteLoops=[[ 62, 2000, 2],[-62, 2000, 2],[ 62, 2000, 2],[-62, 2000, 2],
[ 62, 2000, 2],[-62, 2000, 2],[ 62, 2000, 2],[-62, 2000, 2],
[ 62, 2000, 2],[-62, 2000, 2],[ 62, 2000, 2],[-62, 2000, 2],
[ 62, 2000, 2],[-62, 2000, 2],[ 62, 2000, 2],[-62, 2000, 2],
[ 62, 2000, 2],[-62, 2000, 2],[ 62, 2000, 2],[-62, 2000, 2],
[ 62, 2000, 2],[-62, 2000, 2],[ 62, 2000, 2],[-62, 2000, 3]];
RestartFilename="Entangler";
TSPTau=0.025;
TSPTimeSteps=5;
TSPAdvanceEach=62;
TSPAlgorithm="Krylov";
TSPSites=[1];
TSPLoops=[0];
TSPProductOrSum="sum";
GsWeight=0.1;
string TSPOp0:TSPOperator=expression;
string TSPOp0:OperatorExpression="identity";
```

Above, the `gt1:dir0` connectors give the interactions couplings along the leg direction of the two-leg ladder geometry, so the alternating pattern `[1.0, 0.0, 1.0, 0.0,...]` just means that we evolve in imaginary time the physical sites rather than the ancillas. The imaginary time β' can be obtained with

`h5dump -d /Def/FinalPsi/TimeSerializer/Time <hd5>`, where `<hd5>` should be replaced with the name of the `hd5` file of interest. The targeted β' value is given by $\beta'(J,T) = 1/(2JT)$, where J and T are given in Kelvin. Finally, the arguments to `RecoverySave` mean that we keep a maximum of 100 `hd5` outputs, and output one in every loop (when the condition `1==1` holds). We also observe dipolar as well as quadrupolar correlation functions at finite temperature to extract their characteristic correlation lengths. This is done by doing

```
./observe -f EvolutionT.ain -p 12 "<time|sz;sz|time>"
./observe -f EvolutionT.ain -p 12 "<time|splus*splus;sminus*sminus|time>"
```

Finally, the dynamical spin structure factor is calculated using the operator $\mathcal{L} = H \otimes I + I \otimes (-H)$ with the real space expression in Eq. 2. For the DMRG++ inputs, the dynamics calculation proceeds similarly to the $T = 0$ case, but with number of sites and precision as in the preceding $T > 0$ step. We do, however, need to additionally add/modify the following lines

```
gt0:dir0:Connectors=[1.0, -1.0, 1.0, -1.0, 1.0, -1.0, 1.0, -1.0, ...];
gt0:dir1:Connectors=[0.0, 0.0, 0.0, 0.0, ...];
gt1:dir0:Connectors=[1.0, -1.0, 1.0, -1.0, 1.0, -1.0, 1.0, -1.0, ...];
gt1:dir1:Connectors=[0.0, 0.0, 0.0, 0.0, ...];
RestartFilename="Recovery23EvolutionT.hd5";
SolverOptions="CorrectionVectorTargeting,restart,twositedmrg,minimizeDisk,fixLegacyBugs";
integer RestartSourceTvForPsi=0;
vector RestartMappingTvs=[-1, -1, -1, -1];
integer RestartMapStages=0;
# This is the structure of FiniteLoops for dynamics
FiniteLoops=[[-98, 2000, 2],[98, 2000, 2],[-98, 2000, 2],[98, 2000, 2]];
string TruncationTolerance="1e-7,100";
```

Above, the restart filename should be chosen to match the `hd5` file of interest. All rung couplings are zero. As before, we have abbreviated the arrays of coupling constants.

[1] White, S. R. & Feiguin, A. E. Real-time evolution using the density matrix renormalization group. *Phys. Rev. Lett.* **93**, 076401 (2004).

[2] Nocera, A. & Alvarez, G. Spectral functions with the density matrix renormalization group: Krylov-space approach for correction vectors. *Phys. Rev. E* **94**, 053308 (2016).

[3] Nocera, A. et al. Doping evolution of charge and spin excitations in two-leg Hubbard ladders: Comparing DMRG and FLEX results. *Phys. Rev. B* **97**, 195156 (2018).

[4] Tiegel, A. C., Manmana, S. R., Pruschke, T. & Honecker, A. Matrix product state formulation of frequency-space dynamics at finite temperatures.*Phys. Rev. B* **90**, 060406 (2014).

[5] Feiguin, A. E. & White, S. R. Finite-temperature density matrix renormalization using an enlarged Hilbert space. *Phys. Rev. B* **72**, 220401 (2005).

[6] Feiguin, A. E. & Fiete, G. A. Spectral properties of a spin-incoherent Luttinger liquid. *Phys. Rev. B* **81**, 075108 (2010).

[7] Nocera, A. & Alvarez, G. Symmetry-conserving purification of quantum states within the density matrix renormalization group. *Phys. Rev. B* **93**, 045137 (2016).

[8] White, S. R. & Huse, D. A. Numerical renormalization-group study of low-lying eigenstates of the antiferromagnetic S=1 Heisenberg chain. *Phys. Rev. B* **48**, 3844–3852 (1993).

[9] Horton, M. D. P. & Affleck, I. Three-magnon contribution to the spin correlation function in integer-spin antiferromagnetic chains. *Phys. Rev. B* **60**, 11891–11893 (1999).

[10] Essler, F. H. L. Three-particle scattering continuum in quasi-one-dimensional integer-spin Heisenberg magnets. *Phys. Rev. B* **62**, 3264–3270 (2000).

[11] Xu, G. et al. Y_2BaNiO_5: A nearly ideal realization of the S = 1 Heisenberg chain with antiferromagnetic interactions. *Phys. Rev. B* **54**, R6827–R6830 (1996).

[12] Zaliznyak, I. A., Lee, S.-H. & Petrov, S. V. Continuum in the spin-excitation spectrum of a Haldane chain observed by neutron scattering in $CsNiCl_3$. *Phys. Rev. Lett.* **87**, 017202 (2001).

[13] Xu, G. et al. Mesoscopic phase coherence in a quantum spin fluid. *Science* **317**, 1049–1052 (2007).

[14] Gómez-Santos, G. Variational approach to the xxz spin-1 linear chain: Elementary excitations and haldane conjecture. *Phys. Rev. Lett.* **63**, 790–793 (1989)

[15] Alvarez, G. The density matrix renormalization group for strongly correlated electron systems: A generic implementation. *Comput. Phys. Commun.* **180**, 1572–1578 (2009).

[16] Scheie, A. et al. Witnessing entanglement in quantum magnets using neutron scattering. *Phys. Rev. B* **103**, 224434 (2021).
